# Early postnatal irradiation‐induced age‐dependent changes in adult mouse brain: MRI based characterization

**DOI:** 10.1186/s12868-021-00635-2

**Published:** 2021-04-21

**Authors:** Bo Xu Ren, Isaac Huen, Zi Jun Wu, Hong Wang, Meng Yun Duan, Ilonka Guenther, K. N. Bhanu Prakash, Feng Ru Tang

**Affiliations:** 1 Department of Medical Imaging, School of Medicine, Yangtze University, 1 Nanhuan Road, Jingzhou, 434023 Hubei China; 2 Singapore Bioimaging Consortium (SBIC), Agency for Science, Technology and Research (A*STAR), Singapore, 138667 Singapore; 3 Huaxi MR Research Center (HMRRC), Functional and Molecular Imaging Key Laboratory of Sichuan Province, Department of Radiology, West China Hospital, Sichuan University, Chengdu, China; 4 Radiation Physiology Laboratory, Nuclear Research and Safety Initiative, National University of Singapore, CREATE Tower, 1 CREATE Way #04-01, Singapore, 138602 Singapore; 5 Comparative Medicine, Centre for Life Sciences (CeLS), National University of Singapore, #05-02, 28 Medical Drive, Singapore, 117456 Singapore

**Keywords:** Acute irradiation, Postnatal, MRI, Biomarker, Brain damage, Neurogenesis

## Abstract

**Background:**

Brain radiation exposure, in particular, radiotherapy, can induce cognitive impairment in patients, with significant effects persisting for the rest of their life. However, the main mechanisms leading to this adverse event remain largely unknown. A study of radiation-induced injury to multiple brain regions, focused on the hippocampus, may shed light on neuroanatomic bases of neurocognitive impairments in patients. Hence, we irradiated BALB/c mice (male and female) at postnatal day 3 (P3), day 10 (P10), and day 21 (P21) and investigated the long-term radiation effect on brain MRI changes and hippocampal neurogenesis.

**Results:**

We found characteristic brain volume reductions in the hippocampus, olfactory bulbs, the cerebellar hemisphere, cerebellar white matter (WM) and cerebellar vermis WM, cingulate, occipital and frontal cortices, cerebellar flocculonodular WM, parietal region, endopiriform claustrum, and entorhinal cortex after irradiation with 5 Gy at P3. Irradiation at P10 induced significant volume reduction in the cerebellum, parietal region, cingulate region, and olfactory bulbs, whereas the reduction of the volume in the entorhinal, parietal, insular, and frontal cortices was demonstrated after irradiation at P21. Immunohistochemical study with cell division marker Ki67 and immature marker doublecortin (DCX) indicated the reduced cell division and genesis of new neurons in the subgranular zone of the dentate gyrus in the hippocampus after irradiation at all three postnatal days, but the reduction of total granule cells in the stratum granulosun was found after irradiation at P3 and P10.

**Conclusions:**

The early life radiation exposure during different developmental stages induces varied brain pathophysiological changes which may be related to the development of neurological and neuropsychological disorders later in life.

## Background

Radiotherapy has been used to treat brain tumors and prevent cancer cell metastasis from other organs to the brain, but it induces brain structural and functional changes, which causes lifelong problems with severe societal and economic impact, in particular, in young patients. Brain structural changes such as volume reduction, vascular dilatation and permeability, and white matter pathology ranging from demyelination to coagulative necrosis can be detected by magnetic resonance imaging (MRI) [1]. Functionally, most adults and almost all children who live more than half a year after fractionated radiotherapy have learning and memory problems [2]. Therefore, non-invasive methods, such as MRI or CT scans, are ideal choices to detect brain damage caused due to radiation exposureand to monitor or evaluate the therapeutic effect of radio-neuro-protective drugs during or after radiotherapy or radiation exposure. MRI or CT scans can be used to identify imaging-based biomarkers for radiation-induced cognitive decline or neuropsychological changes. Multi-modal imaging like contrast-enhanced MRI (CE-MRI) and static O-(2-[^18^ F]fluoroethyl)-L- tyrosine (FET) PET radiomics could differentiate radiation injury from recurrent brain metastasis [3]. At the early stage, an acute radiation-induced brain injury could be detected noninvasively by the hybrid multifunctional MRI [4]. Localized brain activity and inter-regional functional connectivity have been studied by resting-state fMRI (rs-fMRI). Using rs-fMRI, early abnormal brain activity could be demonstrated in the surrounding or irradiated brain regions [5]. Hence, in the present study, we aimed to develop MR imaging biomarkers to monitor radiation-induced brain damage and correlate brain neurogenesis alterations to imaging changes and neurological and neuropsychological disorders.

Radio-sensitivity in different brain development stages varies due to differing neurogenesis and gliogenesis; it is, therefore, reasonable to speculate that radiation-induced brain damage varies among animals exposed to different postnatal days. For instance, hippocampal neuronal death peaked at 6 h after X-Ray irradiation at postnatal day 1 with 2 Gy, no cell death was observed at P15 after the irradiation [6–8]. It has been suggested that the mouse brain is most sensitive to neurotoxic injuries including radiation from P7 to P10, as brain growth, gliogenesis, oligodendrocyte maturation, and axonal/dendritic density peak at P10 [9]. However, it remains to be determined which brain regions are more radiosensitive than other regions at each stage of the postnatal brain development period. In the first 2–3 years of human life, the brain is highly radiosensitive due to its rapid development (reaching 90–95% adult brain weight) [7]. The previous studies have suggested that irradiation at the early postnatal days of mice induced the impairment of hippocampal neurogenesis [8, 10, 11], but the long-term effect of irradiation at different postnatal days on the structure of the hippocampus and brain remains unknown. In the present mouse model study, we aimed to investigate and compare the characteristic long-term MRI changes of the early life radiation exposures, i.e., at postnatal day 3 (P3), P10, and P21. The correlation between radiation-induced hippocampal volume changes and neurogenesis was further studied by immunohistochemistry using immature (doublecortin, or DCX), mature (NeuN) neuronal markers, and cell division marker (Ki67).

Brain development in rodents from P1 to P3 is comparable to 23–32 weeks of gestation (preterm infant) in the human [6, 12]. At this stage, the main developmental changes include a predominance of mitotically active preoligodendrocytes[13, 14–16], immune system development [17] and establishment of the blood-brain barrier [18, 19]. From P7 to P10, it is comparable to 36–40 weeks of gestation (preterm infant) in the human [6, 12]. During this period, there are peak brain growth spurt [20, 21] and gliogenesis [22, 23], increasing axonal and dendritic density [24, 25], pre-dominance of immature oligodendrocytes [13–16] and consolidation of the immune system [17], and from P20 to P21, it is comparable to 2–3-year-old children [12]. During this period, rapid brain development, synaptogenesis, myelination, and changes of neurotransmitter and receptor systems occur [6, 12]. In this study, BALB/c mice were chosen because this strain is more radiosensitive than other strains of mice [26, 27] .

## Methods

### Animal irradiation

Neonatal specific-pathogen-free BALB/c mice with mother were provided by InVivos, Singapore, and kept in the Comparative Medicine Facility, the National University of Singapore with free access to water and food in a specific pathogen-free (SPF) facility with room temperature ranging from 210 to 240 °C. Animals were provided with toys and checked by vet daily, and were euthanized with CO2 once they suffered pale mucous membranes, hunched posture and ruffled fur, increased respiratory rate/difficulty breathing, ocular/nasal discharge, unresponsive to gentle prodding, and weight loss > 20% compared to age-matched control.

A total of 28 mice was used in this study. These animals were divided into four groups based on litters, i.e., the normal control group (n = 7, 2 from P3 group, 2 from P10 group, and 3 from P21 group) without irradiation; The experimental groups were whole-body irradiated with 5 Gy (dose rate 2.48 Gy/min) at postnatal day 3 (P3, n = 9), 10 (P10, n = 7), and 21 (P21, n = 5) using the Gamma-Irradiator BIOBEAM8000 (Gamma-Service Medical GmbH, Leipzig, Germany). To monitor the animal radiation dose, two nanoDots dosimeters (LANDAUER, Landauer Global Headquarters, IL, USA) was attached to the opposite sides of the inner wall of the mouse container for each radiation exposure and was read after irradiation using InLight microStar System (LANDAUER, Landauer Global Headquarters, IL, USA) to estimate the actual dose animal received. The actual mean value of nanoDots reading was 0.11 mGy, 4.54 Gy, 4.61 Gy, and 4.77 Gy for the control, P3, P10, and P21 groups respectively. Within thirteen months (13 M) after irradiation, 1, 5, 4, and 2 mice were euthanized in each group of the control, P3, P10, and P21 mice respectively based on the health criteria indicated including (1) weight loss > 20% compared to age-matched control, (2) radiation-induced dermatitis, pale mucous membranes, hunched posture and ruffled fur, increased respiratory rate/difficulty breathing, ocular/nasal discharge, unresponsive to gentle prodding, and (3) skeletal structure extremely prominent, little or no flesh cover and vertebrae distinctly segmented. Euthanization was done by putting animals in a euthanasia chamber, opening the compressed CO2 gas cylinder valve and setting the flow so to displace at least 20% of the chamber volume per minute (usually ~ 5 L/min) to induce rapid unconsciousness with minimal distress to the animals. Gas flow was maintained for at least 1 min after respiration has ceased. We verified that animals were dead before removing it from the chamber by making sure there was no respiratory movement for at least 3 min. If the animal was not dead, cervical dislocation was done following the CO2 administration. Carcass were then stored in −20 °C freezer. MRI scans were therefore performed in 6 (1 male, 5 female), 4 (2 male, 2 female), 3 (1 male, 2 female), and 3 (2 male, 1 female) mice in those respective groups at 13 M after radiation exposure. The previous study indicated that the mean longevity or life-span was 27.2 months in BALB/c female and 21.6 months in BALB/c male mice [28]. We, therefore, chose 13 months after irradiation of P3, P10, and P21 mice as a middle-age (27.2 months/2 = 13.6 months) of animals for MRI scan as the most of animals were female in the present study. The experimental protocols were reviewed and approved by the Institutional Animal Use and Care Committee (IACUC), National University of Singapore (R15-01576).

### Magnetic resonance imaging

#### Acquisition

In vivo imaging was performed at the NUS Comparative Medicine Imaging Facility using a 7 Tesla preclinical animal MRI scanner (Bruker Clinscan, Bruker BioSpin MRI GmbH, Ettlingen, Germany) equipped with a gradient system reaching a maximum amplitude of 400 mT/m. The animals were positioned head first, prone on a mouse bed and the circularly polarized mouse head coil was positioned and fixed covering the head. The animal bed was then movedinto the center of the magnet bore. Anesthesia was maintained with 1.5–2.5% isoflurane in oxygen (flow rate 1 L/min) during image acquisition. Rectal temperature and respiratory rate and pattern were monitored using an MRI-compatible small animal physiological monitoring system (Model 1030, SA Instruments Inc, USA).

For anatomical reference and to check the correct animal alignment in the magnet bore, localizers were acquired in three orthogonal planes: axial, sagittal, and coronal. A 2D Turbo Spin Echo (TSE) protocol (FOV 23mm; matrix size 320 × 320; TR 4400ms; TE 39ms; Flip Angle 180°) was used to produce T2-weighted sagittal images with a slice thickness of 0.4 mm, and an in-plane resolution of 0.072 mm × 0.072 mm. A 3D TSE sequence (FOV 25mm; matrix size 286 × 320; TR 3000ms; TE 121ms; 2 averages) was also used to produce T_2_-weighted coronal images with a slice thickness of 0.2mm and an in-plane resolution of 0.075 mm × 0.078 mm. Each scan lasted approximately 40 min.

#### Analysis

**Fig. 1:**
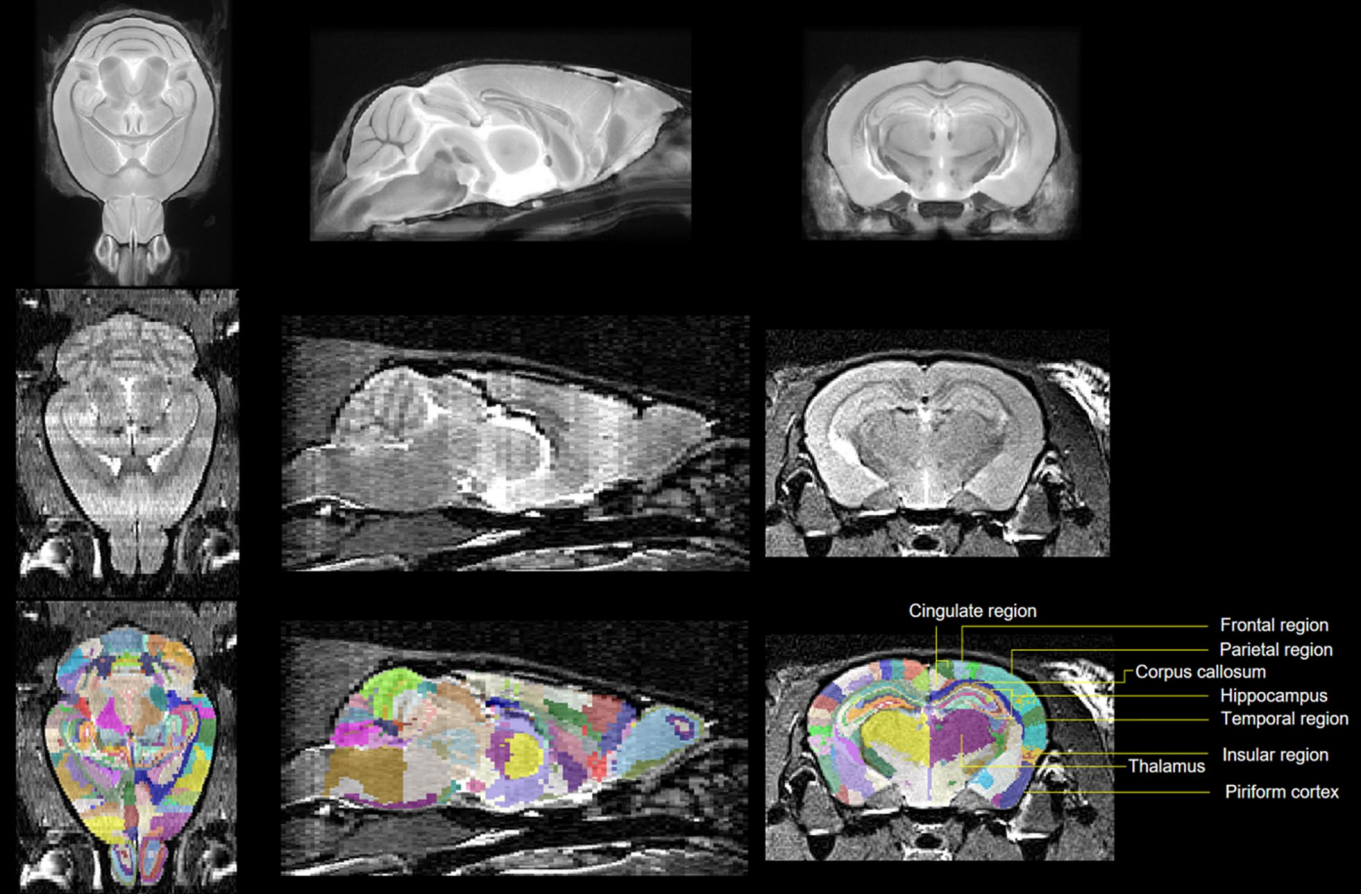
Each subject was segmented by registration of atlas (top row) to subject (middle row) permitting transformation of atlas brain regions onto subject (bottom row)

Three-dimensional (3D) brain images were automatically segmented into 364 labels using an in-house pipeline. This consisted of atlas-based brain extraction [29] followed by atlas-based segmentation [30]. In both cases, the atlas used was the high-resolution mouse brain atlas, whose labels include regions such as CA1 which were assigned to super-regions such as the hippocampus [31, 32]. Brain extraction involved linear registration of atlas brain mask to an estimate of the subject’s brain mask (Brain Extraction Tool) [33]. To verify the accuracy of brain extraction, all registrations were manually reviewed. Segmentation required 3D registration of atlas brain to subject brain using both linear and nonlinear registration, with inspection at every stage (Fig. 1). This allowed the transformation of atlas brain regions onto subject brain regions.

**Fig. 2:**
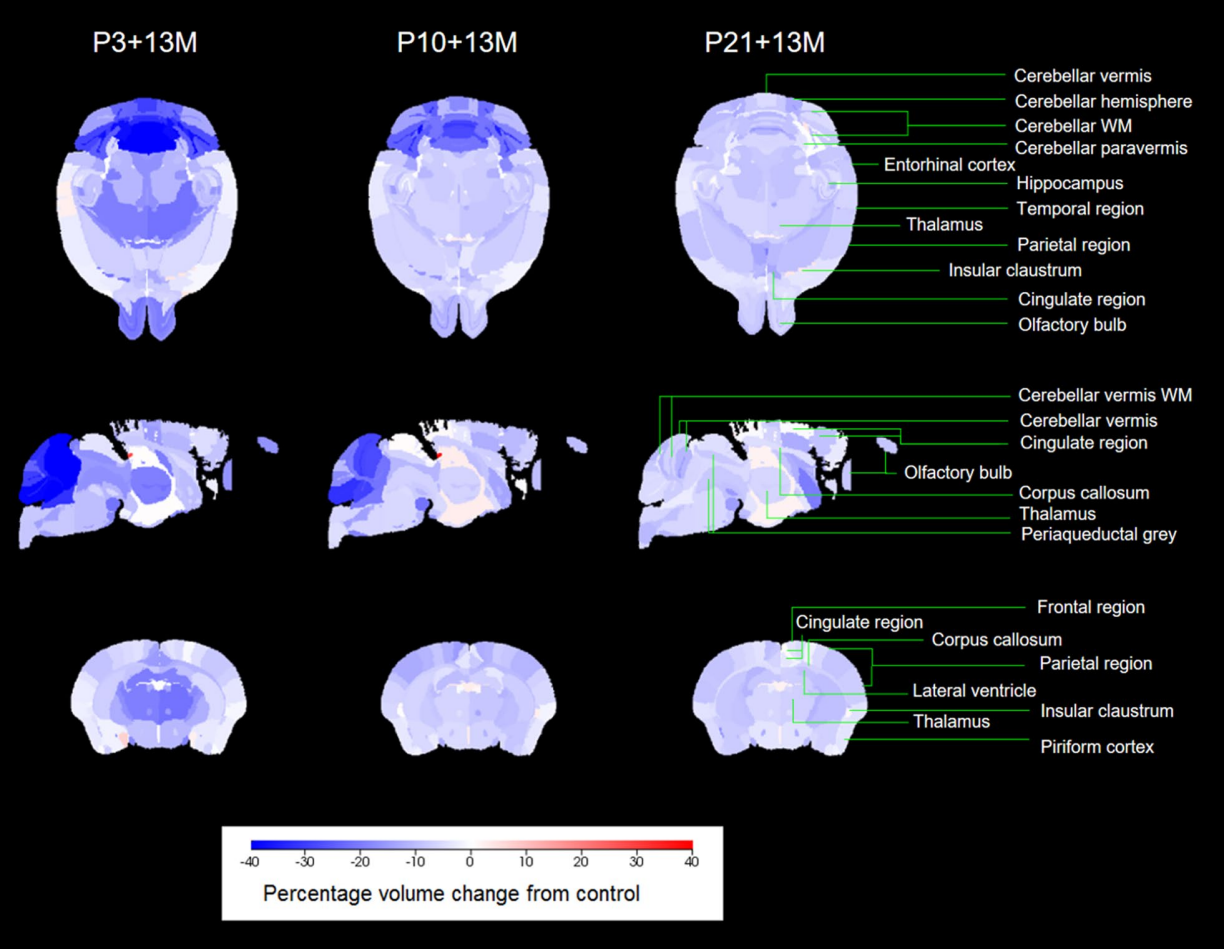
Acute irradiation (5 Gy γ irradiation) induced the percentage volume changes in various parcels of the brain. Compared to the control group, MRI scanning showed that acute gamma radiation (5 Gy γ radiation) caused percentage volume changes in various areas of the brain in the group of P3 + 13 M, P10 + 13 M, P21 + 13 M

**Fig. 3:**
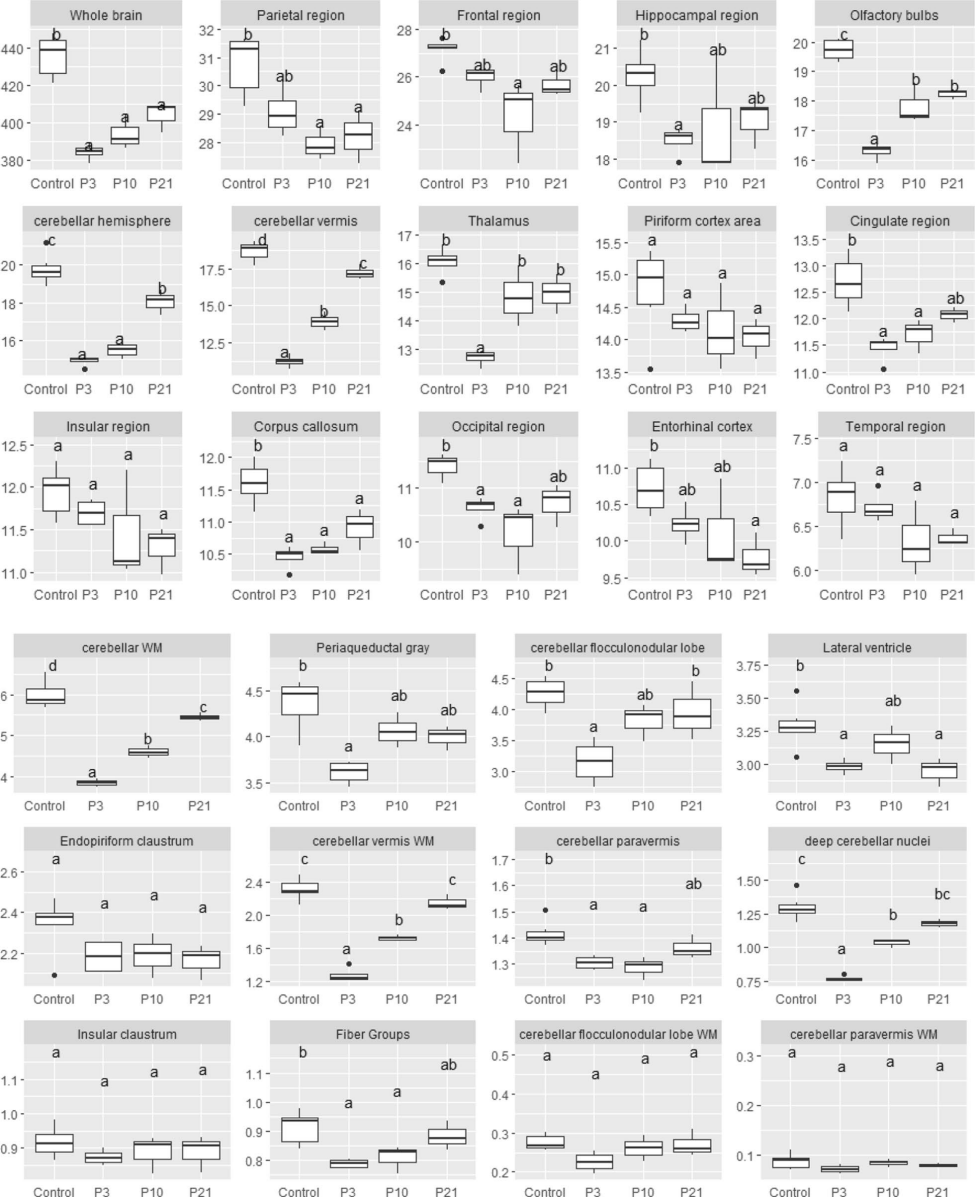
Acute irradiation (5 Gy γ irradiation) induced volume changes in various super-regions of the brain. Compared to the control group (n = 6) MRI scanning shows that acute gamma radiation (5 Gy γ radiation) causes volume changes in various super-regions of the brain in the group of P3 + 13 M (P3) (n = 4), P10 + 13 M (P10) (n = 3), P21 + 13 M (P21) (n = 3). Boxplots show distribution of volumes for each group. Letters denote groups not significantly different from each other. These were calculated by one-way ANOVA between groups followed by calculation of least-squares means (correction for multiple comparisons using Tukey-Kramer test, α = 0.05). Regions are ordered by mean volume of each region

The volume of each region was automatically calculated in mm^3^ using an in-house script developed in the programming language *R*. Percentage differences from the control group were calculated for each region and then converted into a color-coded image (Fig. 2). Volumes of super-regions were calculated by summing regions assigned to them. Comparisons were made between groups for super-regions and selected regions of relevance such as the lateral ventricle (Fig. 3).

**Fig. 4:**
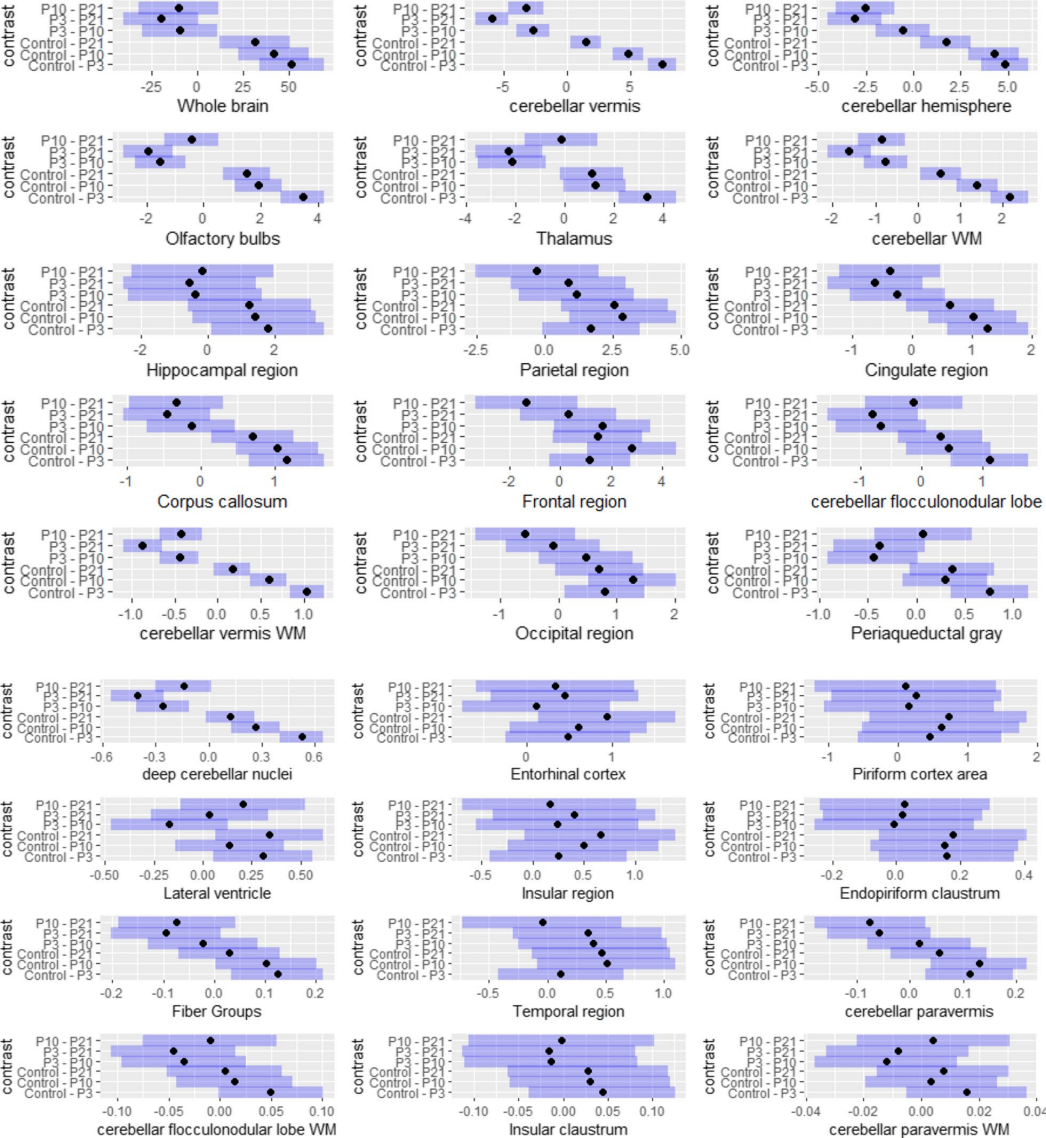
The 95% confidence intervals of volume changes between all groups. These were calculated by one-way ANOVA between groups followed by estimation of least-squares means (correction for multiple comparisons using Tukey-Kramer test, α = 0.05). Dot shows mean and blue bars show standard error. Regions are ordered by descending size of Control – (P3 + 13 M) contrast (**a**, **b**). P3: P3 + 13 M, P10: P10 + 13 M, P21: P21 + 13 M

For each super-region, a boxplot of its volume across groups was plotted and groups without significant differences were labeled by the same letter (Fig. 3). This was achieved by calculation of significance of volume change across all groups using a one-way ANOVA (corrected for an unbalanced design using type III sum of squares), followed by calculation of 95% confidence intervals of volume change (Fig. 4) between individual groups using least-squares means estimation (correction for multiple comparisons using Tukey-Kramer test, α = 0.05). We also investigated the interaction of the group, region, and sex using a three-way ANOVA (corrected for an unbalanced design using type III sum of squares). All statistical analysis was carried out in *R* using the *lsmeans, cars,ggplot2, and gridExtra* packages.

### Immunohistochemistry

At 15 M after irradiation, animals (5 control, 4 P3 + 15 M, 3 P10 + 15 M and 3 P21 + 15 M with the respective mean weight of 32.32, 24.60, 24.57, 25.07 g) were anesthetized with ketamine (75 mg/kg) + medetomidine (1 mg/kg) at 0.1ml/10 g, perfused with saline and fixed with freshly prepared 4% paraformaldehyde solution. The brain was removed, postfixed overnight, and then transferred to 30% sucrose in 0.1 M PB (pH: 7.4). Sagittal sections were then cut at 40 μm thickness, a serial of alternative 6 sections were collected and put into 6 wells. Three wells were processed for immunohistochemistry to investigate radiation-induced changes of cell proliferation, neurogenesis (immature neurons), and mature neurons using Ki67, doublecortin (DCX), or and neuron-specific nuclear protein (NeuN) respectively.

For immunohistochemistry, sections were blocked with 3% H_2_O_2_ for 10 min, followed by 1.5% normal goat serum (for Ki67 and NeuN) or 2% horse serum (for DCX) for 2 h at room temperature. The sections were then treated with primary rabbit antibodies for Ki67 (1: 400) (Gene Tex, USA), and NeuN (1:1500) (Gene Tex, USA) or goat antibodies for DCX (Santa Cruz Biotechnology Inc., CA, USA) in 0.1 M phosphate buffer saline (PBS) with 0.1% Triton X-100 (PBS-TX) overnight. The sections were then treated with biotinylated goat anti-rabbit or horse anti-goat secondary antibodies for 1 h. The sections were then treated with avidin-biotin complex (ABC) reagent (Vector Laboratories Inc., Burlingame, CA, USA) for 1 h, reacted in DAB Peroxidase Substrate (Vector Laboratories Inc., Burlingame, CA, USA) for 10 min, mounted, and covered with a coverslip.

### Cell counting and statistical analysis

Ki67- or DCX- or NeuN-immunostained cells were analyzed by a stereological method using STEREOLOGER^™^ software (Stereological Resource Center Biosciences, Inc. Florida, USA). The experimenter was blind to the group allocation, and only the principal investigator was aware of the group allocation at the different stages of the experiment. “Regional Volume Probe” and “Object Number Probe” were chosen to count positive cell numbers in the entire medial-lateral extent of the dentate gyrus. The section interval was 6. The cell number was indicated as mean ± SEM and was statically analyzed by One-Way ANOVA followed by Tukey’s *post hoc* test. A p-value < 0.05 was considered as statistical significant.

## Results

### Acute γ- irradiation with 5 Gy induced brain volume changes as characterized by MRI scans

Regional percentage volume changes as characterized by MRI scans were visualized in Fig. 2 for P3 + 13 M, P10 + 13 M, and P21 + 13 M groups. For each super-region, the distribution of volumes in each group was shown in Fig. 3. The groups without a significant difference were labeled with the same letter. The 95% confidence intervals of volume changes between individual groups were shown in Fig. 4.

Compared to the control group, the total brain volume was reduced in all three groups of P3 + 13 M (− 51.53 ± 5.88 mm^3^, t-ratio = 8.759, df = 12, p < 0.0001), P10 + 13 M (− 41.74 ± 6.44 mm^3^, t-ratio = 6.476, df = 12, p = 0.0002) and P21 + 13 M (− 31.42 ± 6.44 mm^3^, t-ratio = 4.876, df = 12, p = 0.0019).

Percentage volume change from control ranged from − 45% (cerebellar vermis WM) to − 2% (temporal region, insular region) at P3 + 13 M, − 28% (cerebellar WM) to − 4% (cerebellar paravermis WM, insular claustrum, insular region) at P10 + 13 M and − 9% (deep cerebellar nuclei, cerebellar paravermis WM, cerebellar hemisphere) to − 4% (piriform cortex, insular claustrum, cerebellar paravermis) at P21 + 13 M.

In order, regions with the greatest percentage volume reduction between control and P3 + 13 M groups were cerebellar vermis WM (− 44.7%, − 1.03 ± 0.0653 mm^3^, t-ratio = 15.852, df = 12, p < 0.0001), deep cerebellar nuclei, (− 40.4%, − 0.52 ± 0.0419 mm^3^, t-ratio = 12.504, df = 12, p < 0.0001), cerebellar vermis, (− 39.8%, − 7.44 ± 0.369 mm^3^, t-ratio = 20.155, df = 12, p < 0.0001), cerebellar white matter, (− 35.9%, − 2.15 ± 0.147 mm^3^, t-ratio = 14.623, df = 12, p < 0.0001), cerebellar flocculonodular lobe (− 26.0%, − 1.11 ± 0.211 mm^3^, t-ratio = 5.241, df = 12, p = 0.0001), cerebellar hemisphere (− 24.4%, − 4.82 ± 0.411 mm^3^, t-ratio = 11.729, df = 12, p < 0.0001), thalamus (− 20.9%, − 3.36 ± 0.389 mm^3^, t-ratio = 8.616, df = 12, p < 0.0001), cerebellar paravermis WM (− 18.1%, − 0.0157 ± 0.00705 mm^3^, t-ratio = 2.221, df = 12, p = 0.1727), cerebellar flocculonodular lobe WM (− 18.0%, − 0.0496 ± 0.0173 mm^3^, t-ratio = 2.870, df = 12, p = 0.0593) and olfactory bulbs (− 17.4%, − 3.44 ± 0.25 mm^3^, t-ratio = 13.8, df = 12, p < 0.0001),

Statistical testing of the interaction of group, region and sex using a three-way ANOVA showed significant interactions between group and region (F_1005,2688_ = 6.82, P < 2.2 × 10^− 16^), region and sex (F_335,2688_ = 2.81, P < 2.2 × 10^− 16^) and group and sex (F_3,2688_ = 9.71, P = 2.3 × 10^− 6^). The interaction between region, group and sex was not significant (F_1005,2688_ = 0.88, P = 0.99).

### Acute γ-irradiation with 5 Gy induced impairment of cell proliferation and neurogenesis at 15 M after irradiation

**Fig. 5:**
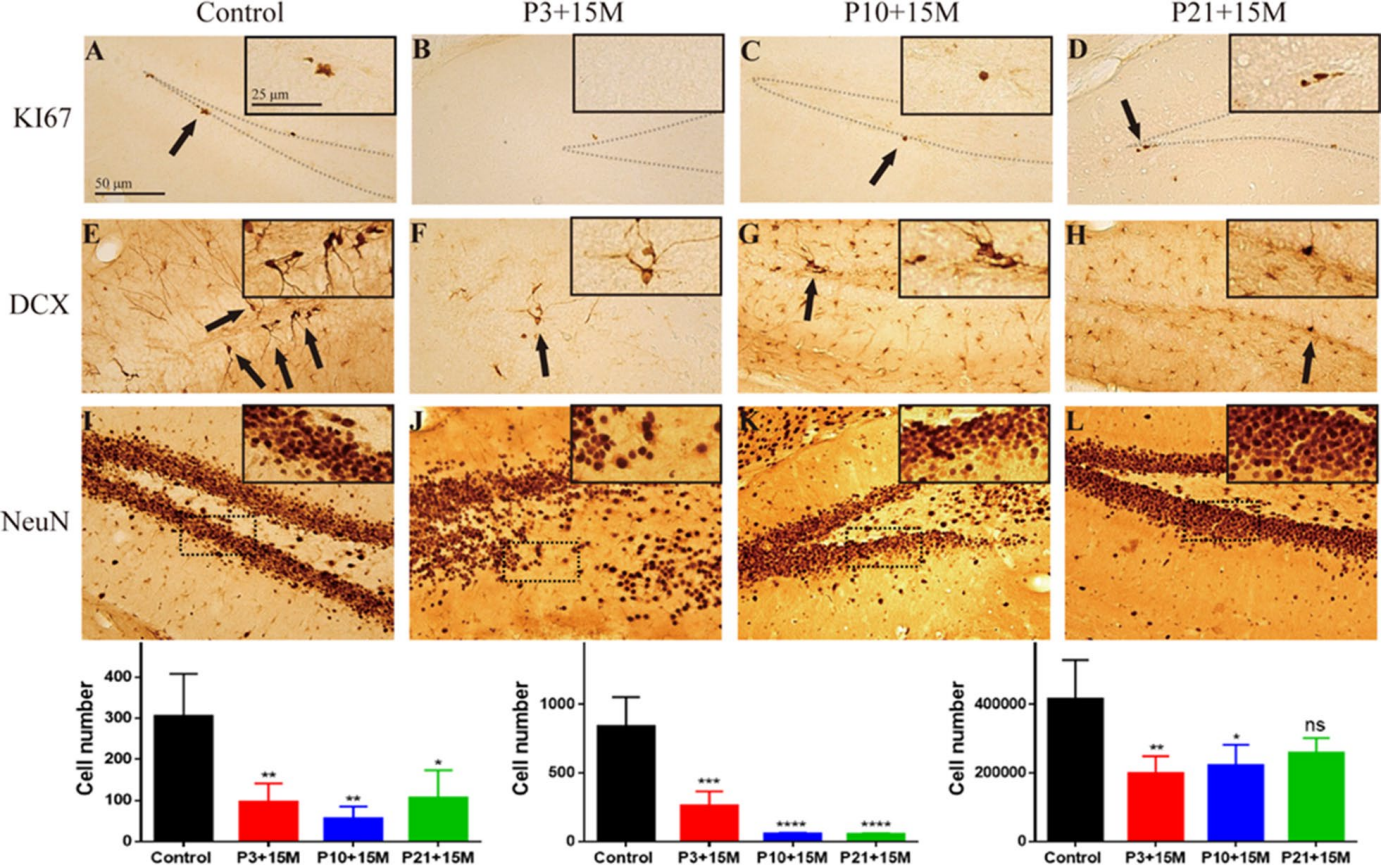
Ki67 immunohistochemistry shows dividing cells (arrows, with high magnification in insert) in the subgranular zone of the dentate gyrus in the control (n = 5) (**a**), P3 + 15 M (with 5 Gy γ-ray irradiation at postnatal day 3, n = 4) (**b**), P10 + 15 M (with 5 Gy γ-ray irradiation at postnatal day 10, n = 3) (**c**), P21 + 15 M (with 5 Gy γ-ray irradiation at postnatal day 21, n = 3) (**d**) mouse respectively. DCX immunohistochemistry shows newly generated neurons (arrows, with high magnification in insert) in the subgranular zone of the dentate gyrus in the normal control (**e**), P3 + 15 M (**f**), P10 + 15 M (**g**), and P21 + 15 M (**h**) group of mice respectively. NeuN immunohistochemistry shows mature neurons in the granule cell layer of dentate gyrus in the normal control (**i**), P3 + 15 M (**j**), P10 + 15 M (**k**), and P21 + 15 M (**l**) group of mice respectively. In P3 + 15 M group, the ventral blade of the stratum granulosum is not fully developed (arrows in **j**) (Insert in **i**–**l** shows magnified NeuN positive cells from the dashline rectangle in each Fig). One-way ANOVA followed by the Tukey’s post hoc test were used for statistical analyses indicate significant reduction of Ki67 (**m**), DCX (**n**) and NeuN (**o**) immunopositive cells. *P < 0.05 compared to the control, **P < 0.01 compared to the control, ***P < 0.001 compared to the control, ****P < 0.0001 compared to the control. ns: P > 0.05 no statistical significant difference compared to the control

The immunohistochemical study of Ki67 (Fig. 5a–d) and DCX (Fig. 5e–h) showed a significant loss of dividing cells (Fig. 5a–d) and newly generated neurons (Fig. 5e–h) in the subgranular zone in P3 + 15 M, P10 + 15 M and P21 + 15 M groups compared to the control. Similarly, NeuN immunohistochemistry (Fig. 5i–l) revealed a significant loss of mature neurons in the stratum granulosum of the dentate gyrus in the group of P3 + 15 M and P10 + 15 M compared to the control, but there is no statistical significance between control and P21 + 15 M. In the P3 + 15 M group, there was hypoplasia of the low blade of the stratum granulosum.

## Discussion

### Main findings

In the present MRI scan, we showed a significant brain region- and age-dependent radiosensitivity after irradiation with 5 Gy. Relative to control, there was a significant reduction at P3 in 17 of the 27 super-regions. At P3, the highest percentage volume reductions were primarily in the cerebellum including its hemispheres, vermis, paravermis, and flocculonodular lobe, although thalamus and olfactory bulbs were also in the top 10. Other notable regions with significant reductions included the hippocampal region, cingulate region, lateral ventricle, and fiber groups.

In many regions, there was a trend of P10 and P21 being less radiosensitive. Both P10 and P21 had significant consecutive increases in cerebellar WM, cerebellar vermis, cerebellar vermis white matter, while at least one of P10 and P21 significantly increased from P3 in olfactory bulbs, thalamus, cerebellar hemisphere, and deep cerebellar nuclei.

At a cellular level, acute irradiation at all the 3 postnatal days reduced dividing cells and newly generated neurons in the subgranular zone of the dentate gyrus indicated by Ki67 and DCX at 15 M after irradiation. Furthermore, the number of NeuN immunostained granule cells in the dentate gyrus was also reduced at 15 M after irradiation when animals were irradiated at P3 and P10.

### Radiation‐induced mouse brain volumetric changes are region- and age‐dependent

Clinical studies suggest that radiation induces the white [34, 35] and gray [11, 36–38] matter atrophies in the human brain [34–45]. These changes were usually induced by very high doses of acute or fractionated radiotherapy and were dose-dependent [36, 41]. It suggests that further study on brain structural changes after irradiation with doses less than 10 Gy is still needed. Irradiation of animals with 5 Gy in the present study may provide a good model for searching MRI markers for detection of relatively low dose radiation-induced brain pathophysiological changes.

Radiation-induced brain damage is age- and region-dependent. Cortical thinning in the pediatric population with radiotherapy for medulloblastoma was found predominantly in those brain regions undergoing active development which are therefore more radiosensitive than other regions [46]. In adult patients with high-grade glioma (HGG), the temporal lobe was the most radiosensitive neuroanatomic location at 1-year post-radiotherapy [38]. However, it remains unknown whether the minor age difference among the pediatric population will be related to radiation-induced brain damage in different brain regions. In the present mouse model, we showed significant volume changes in the hippocampus, and cerebellar flocculonodular lobe when animals were irradiated at P3, but not P10 and P21, suggesting that radiation exposure at different early postnatal days may induce varied brain pathological changes and subsequent neurological and neuropsychological disorders in the later stages of animal or human life.

While most mouse brain neurogenesis occurs prenatally, neurogenesis in several brain regions including granule cells of the dentate gyrus in the hippocampus, granule cells of the olfactory bulbs, and the cerebellar cortex undergo their principal development during the postnatal period, in particular, in the first 1 or 2 weeks of postnatal neurodevelopment in rodents [47]. In the present study in the mouse model, several relevant regions had significant volume reduction at P3, but not at one or both of P10 and P21, including the hippocampus and some cerebellar regions including cerebellar paravermis, deep cerebellar nuclei, and cerebellar flocculonodular. This suggests volume reduction is closely related to the postnatal brain developmental stages.

The radiosensitivity of adult brain tumor patients is also brain function-dependent. Brain regions with sophisticated thinking skills such as the entorhinal cortex and the lateral inferior parietal cortex are more vulnerable than the primary visual cortex or primary somatosensory/motor cortex to radiation-induced damage. It was supported by the clinical finding that patients with radiotherapy had neurocognitive impairment [36].

In our mouse model, we have also shown the loss of spatial memory and induced depression when animals were irradiated at P3 which may be related to the impairment and aberrant neurogenesis in the dentate gyrus [48]. The current Ki67 and DCX immunostaining at 15 months after irradiation of P3 mice further confirmed our previous study.

### Radiation‐induced neuropathological changes in different brain regions

#### Hippocampus

Radiation-induced pathophysiological changes in the hippocampus have been well documented [8, 49]. Acute irradiation of immature mice at P0 (6 Gy to the mouse brain) [50], P9 (6 Gy to the mouse brain) [51], P10 (6 or 12 Gy to the mouse brain) [52], P11 (8 Gy to the mouse brain) [53], P14 (8 Gy to the mouse brain) [54, 55], P21 (5 Gy to the mouse brain) [56] and P30 (15-25 Gy to the rat brain) [49, 57] induced impairment of hippocampal neurogenesis when animals were tested at mature ages. Clinical MRI studies indicated that radiotherapy may cause dose-dependent hippocampal atrophy, suggesting a potential of using this biomarker to constrain radiotherapy dose in order to prevent its subsequent cognitive impairment [40–44, 58, 59]. In the present study, hippocampal atrophy occurred after irradiation with a relative low dose of 5 Gy at P3, but not P10 and P21, suggesting that mouse hippocampus is more radiosensitive at P3 than P10 and P21. The immunohistochemical study indicated significant reduction of cell division and newly generated neurons in the dentate gyrus 15 months after irradiation at P3, P10 and P21. The number of mature neurons in the stratum granulosum was also reduced after irradiation at P3 and P10. The impairment of neurogenesis may lead to reorganized neural circuits which may be involved in radiation-induced different neurological and neuropsychological disorders.

#### Cerebrum

Brain MRI study has also shown open-lip schizencephaly with an absence of considerable portions of the right frontal, parietal, and temporal lobes in a patient prenatally exposed to the Chernobyl nuclear disaster after the 28th week of pregnancy [60]. While the authors concluded that it was unlikely that radiation could account for the anatomic abnormality [60], a constellation of clinical neurophysiological examination and neurobehavioral tests strongly suggests that preterm radiation exposure induces extensive brain damage [61, 62]. Prophylactic whole-brain radiation therapy of acute lymphoblastic leukemia (ALL) at age 5 of a girl induced different neurological and neuropsychological disorders at the late stages of her life [63]. In the present study, the highest whole brain volume reduction was observed after irradiation at P3 which was followed by P10 and P21. The radiation-induced whole brain volume reduction in the mouse model confirms recent clinical studies showing whole-brain volume changes following radiotherapy in either child [64] or adult [65] patients.

#### Cerebellum

The fastest development of the human cerebellum occurs from 24 to 40 weeks of gestation [66, 67]. Harmful environmental exposures during pregnancy, in particular in the fastest development period have been reported to induce abnormal prenatal cerebellar development [68, 69]. The structural changes in the subcortical and cortical areas may also affect vestibular and acoustic functions in clean-up workers of the Chernobyl accident (30 years of follow-up) [70]. In the present study, P3 mice are comparable to the human fetal period of 23–32 weeks of gestation. Since a rapid growth of the cerebellum occurs at this postnatal age, this may explain why the cerebellum is in general more radiosensitive than other brain structures. Our results matched with a previous study [71] showing abnormal cytoarchitecture of the cerebellar cortex and motor abnormalities after a single dose X-irradiation (5 Gy) of rats immediately after birth. It also confirmed the cerebellar regional variation in radiosensitivity after X-ray irradiation of P1 rat with 1.5 Gy and examined at 3 weeks of age [72]. At cellular levels, radiation-induced progenitor cell death in the external germinal layer, microglial reaction, and impairment of the blood-brain barrier may be involved in the volume reduction or atrophy of the cerebellum [73].

#### Olfactory bulb

Radiation exposure also affects the olfactory system. Acute irradiation of immature (P19) or mature (8–12 weeks) mice with high doses of 7.5 or 7 Gy respectively reduced both neurogenesis and volume of the olfactory bulb (OB) [74, 75]. Fractionated irradiation also caused atrophy of OB which was indicated by MRI [76–78]. At a molecular level, P53 mediated apoptosis plays an important role in volume loss in the hippocampus and OB after radiation exposure [79]. Radiotherapy of patients with head and neck cancer also reduced peripheral progenitor cell numbers and taste dysfunction which may persist for months and often years after treatment [80, 81]. In the present study, significant atrophy of OB was observed at P3, with reduced but still significant atrophy at P10 and P21, after radiation exposure with 5 Gy.

#### Thalamus

The brain neuropathological changes in different neurological and neuropsychological disorders varied. Delli Pizzi et al. [82] reported structural connectivity alterations between cortical and subcortical regions or within a cortical network in dementia with Lewy body (DLB), whereas disconnection of mnemonic pathways was present in AD [82]. In DLB, the closed match of neuropathological changes in grey and white matter suggests that neuronal loss but not white matter damage may be related to disruption of brain connectivity. Both cortical and subcortical neuropathological changes are involved in schizophrenia. X-ray irradiation (with a total dose of 1.75–3.5 Gy) of macaques in early gestation but not midgestation reduced thalamic neuron numbers, in particular in the mediodorsal nucleus (MD). Early prenatal radiation exposure-induced neuronal loss in the thalamus results in the reduction of cortical volume, which may imitate the thalamocortical pathology of schizophrenia [83, 84]. Irradiation in adulthood caused behavioral abnormalities relevant to schizophrenia, and reduction of adult neurogenesis by irradiation may be associated with schizophrenia-like behaviors in rats [85]. In the present study, a significant reduction of thalamic volume occurred after irradiation at P3 but not P10 and P21, combined with atrophy of cortex and reduced neurogenesis in the subgranular zone. It is speculated that schizophrenia may be developed at certain stages of animal life after irradiation at P3.

In patients with epilepsy and intellectual disability, cortical dysplasia has been reported, it may be closely linked to clinical manifestation [86]. The atrophy of the animal brain after irradiation at P3, P10, and P21 may also induce epilepsy and intellectual disability which is supported by previous human epidemiological and animal experimental studies [87–89].

In the present study, both male and female mice were used as we kept litters with dams. While different brain regions are sexually dimorphic in terms of volume [90], volume, and time course of development [91], this study only reported the difference in mature mice age 84 days [90], or immature stages from P3 to P65 without any brain insults (normal development) [91]. No evidence was provided regarding the difference at the late stages of animal life. Furthermore, the brain insults at the early stages of animal life may eliminate those changes as the previous studies by pre-or post-natal irradiation of rodents did not show obvious differences in radiosensitivity between the sexes or tested strains. For instance, Jensh [92] reported significant sex differences in responses within the prenatally irradiated and control groups for behavioral tests, but these changes were unrelated to radiation exposure. Eriksson et al. [93] showed an altered adult spontaneous behavior and impaired habituation capacity after irradiation of mice at P3 and P10 but did not find a major difference in neurobehavioural defects between male and female mice, in neither Naval Medical Research Institute (NMRI) mice nor C57BL/6, suggesting no obvious differences in susceptibility between the sexes or tested strains. Furthermore, in these studies, C57BL/6 mice were used, which are different from BALB/c mice used in the current study. Our statistical testing of the interaction among the region, group, and sex did not show a significant difference.

## Conclusions

Both epidemiological and animal experimental studies have suggested the link between prenatal or postnatal radiation exposure and different neurological and neuropsychological disorders. In the present MRI study, characteristic regional volume changes were observed at 13 months after irradiation at P3, P10, and P21, suggesting different brain disorders may be developed at later stages of animal life. These characteristic regional volume change patterns may be used as biomarkers to retrospectively calculate the radiation doses animals are exposed at the early stages of their life. From this point of view, non-invasive MRI scanning may provide morphological biomarkers for retrospective estimation of the radiation doses animals may be exposed in addition to prospective monitoring of radiation-induced brain damage from both radiotherapy and accidental radiation exposure. Further study in patients with radiotherapy or radiodiagnostic exposure may provide more evidence for the development of MRI biomarkers for radiation exposure detection.

## Data Availability

The datasets generated and/or analyzed during the current study are not publicly available but will provide if required by researchers. The study data are available when required.
